# Can Fasting Glucose Levels or Post-Breakfast Glucose Fluctuations Predict the Occurrence of Nocturnal Asymptomatic Hypoglycemia in Type 1 Diabetic Patients Receiving Basal-Bolus Insulin Therapy with Long-Acting Insulin?

**DOI:** 10.1371/journal.pone.0144041

**Published:** 2015-12-01

**Authors:** Sumie Mitsuishi, Rimei Nishimura, Kiyotaka Ando, Daisuke Tsujino, Kazunori Utsunomiya

**Affiliations:** 1 Division of Diabetes, Metabolism and Endocrinology, Department of Internal Medicine, Jikei University School of Medicine, Tokyo, Japan; 2 Graduate School of Public Health, University of Pittsburgh, Pittsburgh, Pennsylvania, United States of America; Weill Cornell Medical College Qatar, QATAR

## Abstract

**Objective:**

To investigate whether the occurrence of nocturnal asymptomatic hypoglycemia may be predicted based on fasting glucose levels and post-breakfast glucose fluctuations.

**Patients and Methods:**

The study subjects comprised type 1 diabetic patients who underwent CGM assessments and received basal-bolus insulin therapy with long-acting insulin. The subjects were evaluated for I) fasting glucose levels and II) the range of post-breakfast glucose elevation (from fasting glucose levels to postprandial 1- and 2-hour glucose levels). The patients were divided into those with asymptomatic hypoglycemia during nighttime and those without for comparison. Optimal cut-off values were also determined for relevant parameters that could predict nighttime hypoglycemia by using ROC analysis.

**Results:**

64 patients (mean HbA1c 8.7 ± 1.8%) were available for analysis. Nocturnal asymptomatic hypoglycemia occurred in 23 patients (35.9%). Fasting glucose levels (I) were significantly lower in those with hypoglycemia than those without (118 ± 35 mg/dL vs. 179 ± 65 mg/dL; *P* < 0.001). The range of post-breakfast glucose elevation (II) was significantly greater in those with hypoglycemia than in those without (postprandial 1-h, *P* = 0.003; postprandial 2-h, *P* = 0.005). The cut-off values determined for relevant factors were as follows: (I) fasting glucose level < 135 mg/dL (sensitivity 0.73/specificity 0.83/AUC 0.79, *P* < 0.001); and (II) 1-h postprandial elevation > 54 mg/dL (0.65/0.61/0.71, *P* = 0.006), 2-h postprandial elevation > 78 mg/dL (0.65/0.73/0.71, *P* = 0.005).

**Conclusions:**

Nocturnal asymptomatic hypoglycemia was associated with increases in post-breakfast glucose levels in type 1 diabetes. Study findings also suggest that fasting glucose levels and the range of post-breakfast glucose elevation could help predict the occurrence of nocturnal asymptomatic hypoglycemia.

## Introduction

The goal of diabetes treatment is to prevent the onset of diabetic complications and to ensure quality of life (QOL) and longevity comparable to those in healthy individuals by controlling glucose and metabolism. HbA1c, a typical index for glucose control, reflects mean glucose levels that vary over the long term. However, large-scale clinical studies found that lowering HbA1c levels does not necessarily result in improved life prognosis [1–3].

Moreover, glucose control aimed at HbA1c reductions may be associated with increased risk of hypoglycemia [4]. A sub-analysis of the ADVANCE study reported that serious hypoglycemia is a determinant of prognosis in cardiovascular and overall mortality [5]. Also, hypoglycemia is reported to induce arrhythmia [6], especially in nocturnal hypoglycemia leading to increased sympathetic nerve activity, followed by over-compensatory vagal activity, thus causing life-threatening brachycardia [7]. It is therefore suggested that nocturnal hypoglycemia may be associated with the so-called “dead in bed” syndrome or sudden death [8–11].

In fact, by using continuous glucose monitoring (CGM), several reports indicated that type 1 diabetic patients are frequently associated with nocturnal hypoglycemia [12–14].

Thus, it appears critically important to predict the occurrence of hypoglycemia and to improve HbA1c while avoiding hypoglycemia as much as possible. Given that hypoglycemia may remain asymptomatic, however, CGM may be required to detect the occurrence of nocturnal hypoglycemia in all patients.

The Somogyi effect is used to describe how fasting glucose levels may become elevated by counter-regulatory hormones in response to nocturnal asymptomatic hypoglycemia [15]. The dawn phenomenon, defined as increased fasting glucose levels (resulting from an insufficiency of basal insulin and a surge of counter-regulatory hormones), is also used to account for elevated fasting glucose. It is difficult to determine without using CGM, however, whether fasting glucose levels become elevated through the Somogyi effect or the dawn phenomenon. Furthermore, while nocturnal hypoglycemia has been reported to elevate post-breakfast glucose levels in patients treated with continuous subcutaneous insulin infusion (CSII) [16], to date, very few studies examined how nocturnal hypoglycemia may affect fasting and post-breakfast glucose levels in patients receiving basal-bolus insulin therapy.

In this study, therefore, type 1 diabetic patients receiving basal-bolus insulin therapy with long-acting insulin were assessed by CGM to examine whether the occurrence of nocturnal asymptomatic hypoglycemia may be predicted based on their fasting glucose levels and post-breakfast glucose fluctuations.

## Patients and Methods

The study subjects comprised type 1 diabetic patients who were admitted to our hospital, assessed by CGM for their glucose profiles and received basal-bolus insulin therapy with long-acting insulin (insulin glargine or insulin detemir). For analysis, CGM data were continuously recorded by using CGMS Gold from immediately after admission onwards for at least 24 hours. Then, patients were excluded from analysis if they had taken oral glucose upon becoming aware of nocturnal hypoglycemia and if they had taken oral α-glucosidase inhibitors, which are known to affect postprandial glucose levels.

Nocturnal asymptomatic hypoglycemia was defined as hypoglycemia (< 70 mg/dL) occurring between 12 pm and 6 am and the subjects were evaluated for 1) nocturnal glucose nadirs and 2) duration of nocturnal asymptomatic hypoglycemia. The subjects were also evaluated for I) fasting glucose levels; II) post-breakfast glucose levels (peak levels as well as postprandial 1- and 2-hour levels); III) the range of post-breakfast glucose elevation (from fasting glucose levels to peak, postprandial 1- and 2-hour glucose levels); and IV) the post-breakfast glucose concentration gradient (from fasting glucose levels to peak, postprandial 1- and 2-hour glucose levels). The subjects were divided into hypoglycemic and non-hypoglycemic patients and compared for parameters I) through IV) by using t-test to examine whether the occurrence of nocturnal asymptomatic hypoglycemia may be predicted based on analysis of parameters I) through IV). A receiver operating characteristic (ROC) analysis was also conducted to determine appropriate cut-off values for these parameters. Multivariate logistic regression was also conducted to identify predictors of nocturnal asymptomatic hypoglycemia.

As this retrospective study used anonymized patient data obtained in routine clinical settings, informed consent was not obtained from the patients. The study protocol was approved by the ethics committee of the Jikei University School of Medicine.

## Results

Of all patients who underwent CGM assessments during hospitalization, 71 were type 1 diabetic patients receiving basal-bolus insulin therapy. Of these, those who had taken oral glucose upon becoming aware of nocturnal hypoglycemia (n = 4) and those who had taken oral α-glucosidase inhibitors (n = 3) were excluded from analysis. As a result, 64 patients were available for analysis in this study (males/females, 20 [31.3%]/44 [68.8%]; mean age, 43.1 ± 14.4 years old; mean disease duration, 15.7 ± 11.6 years; HbA1c, 8.7 ± 1.8%; BMI, 22.2 ± 3.4 kg/m^2^; and urinary C-peptide, 3.6 ± 6.0 μg/day) (Table 1, S1 File). Basal insulin was used once in 15 patients (23.4%) in the evening or before bedtime or twice in 49 patients (76.6%), i.e., once in the morning and once in the evening or before bedtime. Of the two basal insulin preparations used, insulin glargine was used in 35 patients (54.7%) and insulin detemir in 29 patients (45.3%).

**Table 1:** Patient Profile and Parameters for Glycemic Variability Compared between Hypoglycemic and Non-hypoglycemic Patients.

	Overall	Hypoglycemic	Non-hypoglycemic	*P* value*
Patients tested (n)	64	23	41	
Age (years)	43.1 ± 14.4	42.9 ± 14.3	43.2 ± 14.7	*P* = 0.937
HbA1c (%)	8.7 ± 1.8	8.3 ± 1.7	8.9 ± 1.9	*P* = 0.251
Body Mass Index (kg/m^2^)	22.4 ± 3.4	21.6 ± 2.8	22.9 ± 3.7	*P* = 0.147
Urinary C-peptide (μg/day)	3.6 ± 6.0	4.5 ± 7.7	3.0 ± 4.8	*P* = 0.417
Duration of diabetes (years)	15.7 ± 11.6	12.6 ±11.7	17.2 ± 11.4	*P* = 0.141
Total daily insulin dose (TDD) (U/kg)	0.71±0.76	0.93±1.22	0.58±0.16	*P* = 0.184
Basal insulin ratio (%)	43.4 ± 12.7	43.3 ± 13.5	43.4 ± 12.4	*P* = 0.977
Nighttime glucose nadir levels (mg/dL)	98 ± 48	51 ± 11	124 ± 40	*P* < 0.001
Nighttime duration of hypoglycemia (min)	64 ± 107	177 ± 110	0 ± 0	*P* < 0.001
Fasting glucose levels (mg/dL)	157 ± 63	118 ± 35	179 ± 65	*P* < 0.001
Post-breakfast glucose levels (mg/dL)				
Peak	240 ± 58	225 ± 59	249 ± 56	*P* = 0.116
Postprandial 1-h	203 ± 58	186 ± 53	213 ± 59	*P* = 0.072
Postprandial 2-h	207 ± 60	202 ± 66	210 ± 56	*P* = 0.634
Range of post-breakfast glucose elevation (mg/dL)				
Peak	84 ± 53	107 ± 50	70 ± 50	*P* = 0.006
Postprandial 1-h	46 ± 45	67 ± 33	34 ± 46	*P* = 0.003
Postprandial 2-h	50 ± 74	84 ± 54	31 ± 77	*P* = 0.005
Post-breakfast glucose concentration gradient (mg/dL/min)				
Peak	0.91 ± 0.83	1.05 ± 0.35	0.83 ± 1.00	*P* = 0.319
Postprandial 1-h	0.77 ± 0.75	1.13 ± 0.56	0.57 ± 0.77	*P* = 0.003
Postprandial 2-h	0.42 ± 0.62	0.70 ± 0.45	0.26 ± 0.64	*P* = 0.005

Data are shown as mean ± SD

*t-test was employed for comparisons between the hypoglycemic and non-hypoglycemic patients.

The subjects (n = 64) were shown to have 1) a nocturnal glucose nadir of 98 ± 48 mg/dL, and 2) nocturnal asymptomatic hypoglycemia lasting 64 ± 107 minutes. The results for the parameters for post-breakfast glucose fluctuations were as follows: I) fasting glucose levels, 157 ± 63 mg/dL; II) post-breakfast peak, 1- and 2-hour glucose levels, 240 ± 58 mg/dL, 203 ± 58 mg/dL and 207 ± 60 mg/dL; III) range of post-breakfast peak, postprandial 1- and 2-hour glucose elevation, 84 ± 58, 46 ± 45 and 50 ± 74 mg/dL; and IV) post-breakfast peak, postprandial 1- and 2-hour glucose concentration gradient, 0.91 ± 0.83, 0.77 ± 0.75, 0.42 ± 0.62 mg/dl/min (Table 1).

**Fig 1:**
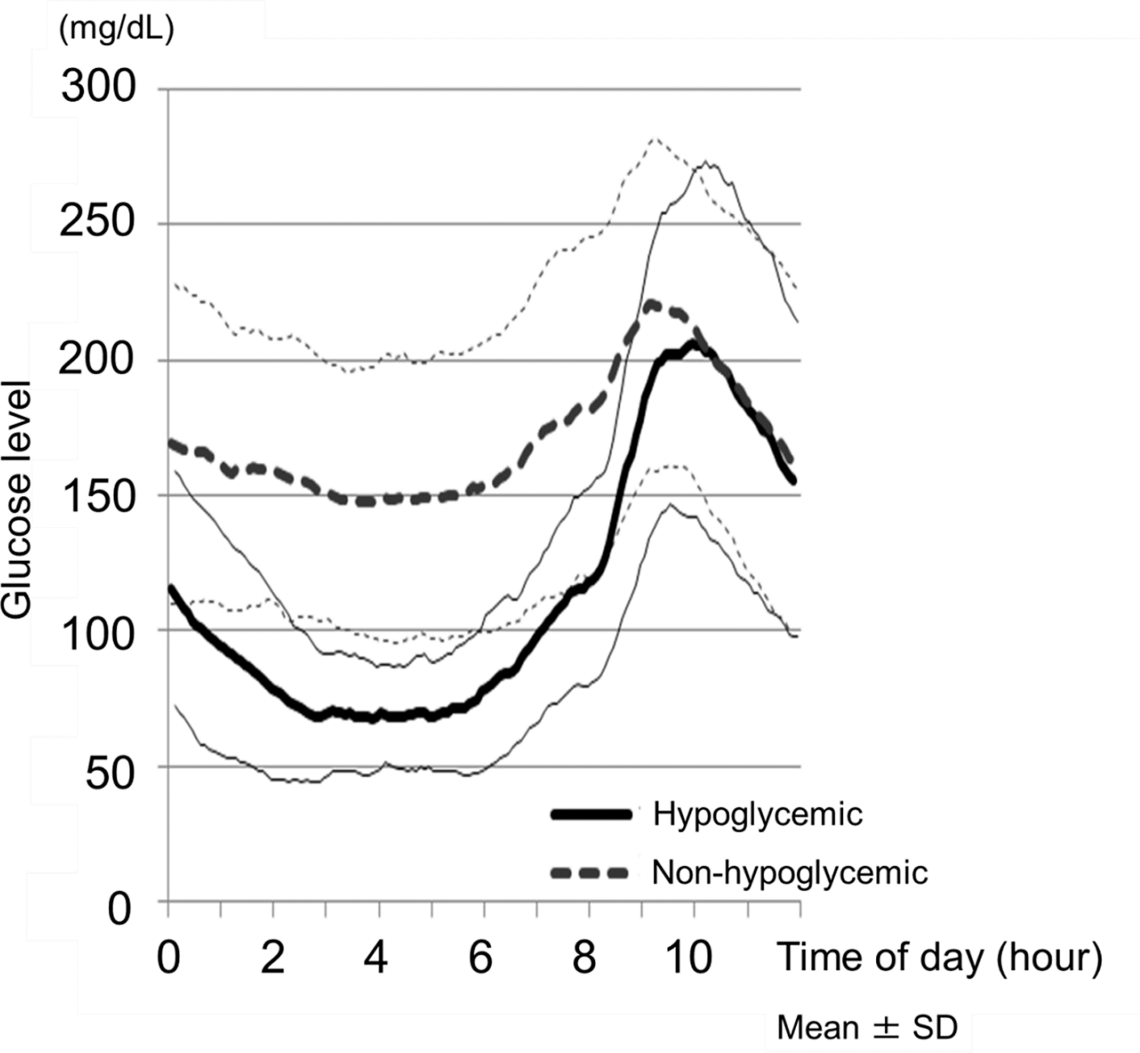
Glucose Profiles Showing Nighttime to Post-Breakfast Glucose levels. Hypoglycemic patients (n = 23); Non-hypoglycemic patients (n = 41).

Nocturnal asymptomatic hypoglycemia occurred in 23 patients (35.9%). Mean HbA1c did not differ between those with hypoglycemia and those without (Table 1). No significant difference was observed between the 6 patients (40.0%) receiving once daily insulin (in the evening or before bedtime) and the 17 patients (34.7%) receiving twice daily insulin (once in the morning and once in the evening or before bed) with regard to the frequency of nocturnal asymptomatic hypoglycemia experienced due to the difference in the timing of basal insulin between the two groups (P = 0.71; χ^2^ test). Glucose profiles from nighttime to post-breakfast hours are shown for those with hypoglycemia and those without in Fig 1. Table 1 summarizes the results for the parameters examined for post-breakfast glucose variability in those with hypoglycemia versus those without. The fasting glucose levels were significantly lower among those with hypglycemia at 118 ± 35 mg/dL compared to 179 ± 65 mg/dL among those without (P < 0.001). On the other hand, the post-breakfast peak, postprandial 1- and 2-hour levels were not significantly different between the two groups. However, the range of glucose elevation at postprandial 1 and 2 hours was significantly greater among those with hypoglycemia (postprandial 1-hour, 67 ± 33 vs. 34 ± 46 mg/dL, P = 0.003; postprandial 2-hour, 84 ± 54 vs. 31 ± 77 mg/dL, P = 0.005), as was the post-breakfast glucose concentration gradient (postprandial 1-hour, 1.13 ± 0.56 vs. 0.57 ± 0.77 mg/dL/min, P = 0.003; postprandial 2-hour, 0.70 ± 0.45 vs. 0.26 ± 0.64 mg/dL/min, P = 0.005).

**Fig 2:**
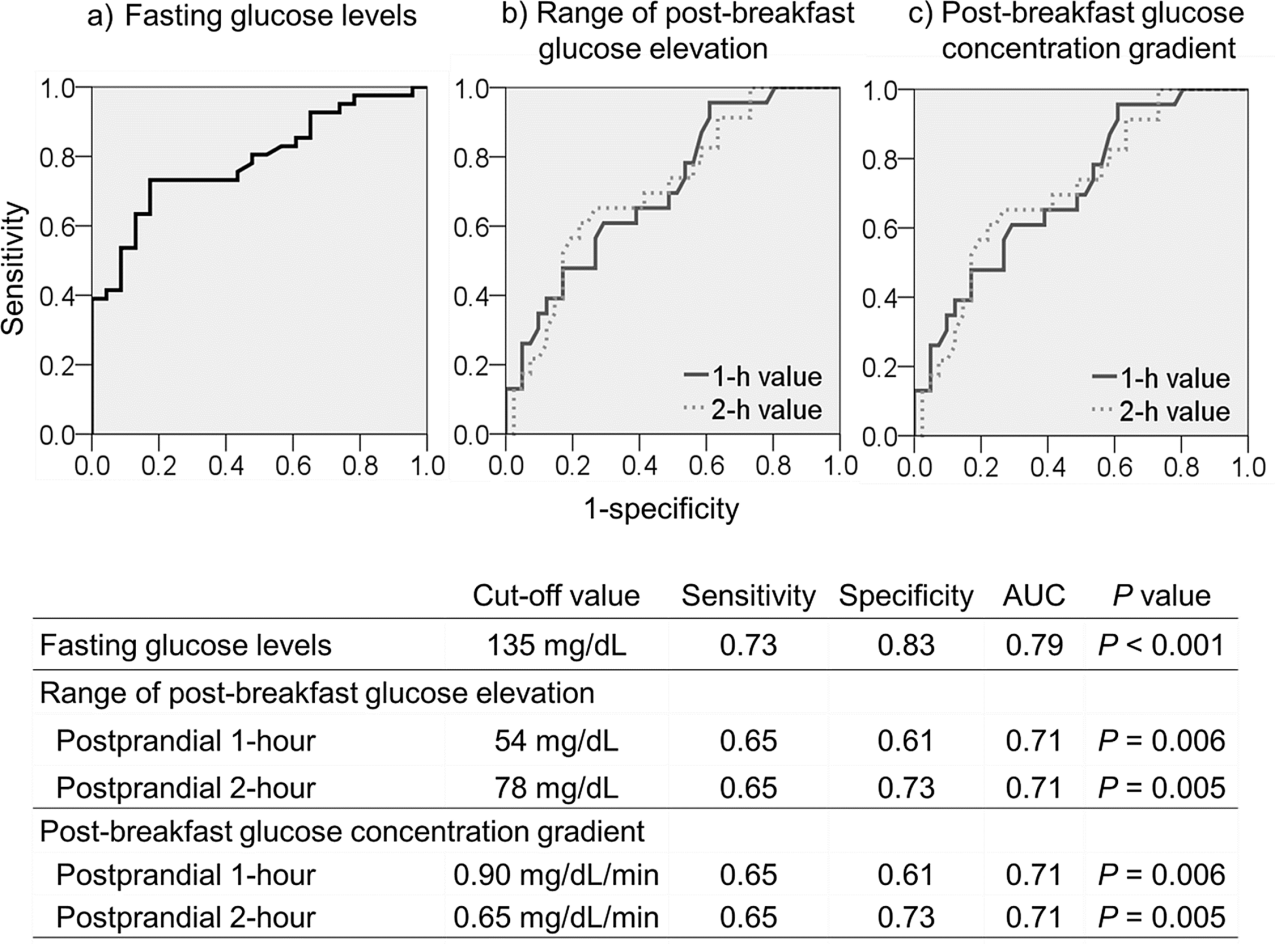
Cut-off Values for Predicting Nocturnal Asymptomatic Hypoglycemia.

The ROC analysis-derived cut-off values for prediction of nocturnal asymptomatic hypoglycemia were as follows: fasting glucose levels, < 135 mg/dL (sensitivity 0.73/specificity 0.83/AUC 0.79, P < 0.001); range of post-breakfast glucose elevation, > 54 mg/dL at postprandial 1 hour (0.65/0.61/0.71, P = 0.006); > 78 mg/dL at postprandial 2 hours (0.65/0.73/0.71, P = 0.005); and post-breakfast glucose concentration gradient, > 0.90 mg/dL/min at postprandial 1 hour (0.65/0.61/0.71, P = 0.006), > 0.65 mg/dL/min at postprandial 2 hours (0.65/0.73/0.71, P = 0.005) (Fig 2).

The logistic regression model identified the following factors as predictors of nocturnal asymptomatic hypoglycemia (each shown with its adjusted OR and 95% confidence interval): HbA1c (per 1% increase in HbA1c), 0.46, 0.25–0.84; total daily dose of insulin (per 1 U/kg increase in total daily dose of insulin), 75.1, 1.65–3418; and insulin glargine (vs. insulin detemir), 6.93, 1.42–34.0. The logistic regression model suggested, however, that the following did not predict the occurrence of nocturnal asymptomatic hypoglycemia: female sex (2.35, 0.48–10.6); BMI (per 1 kg/m2 increase in BMI) (1.00, 0.79–1.25); urinary C-peptide (per 1 μg/day increase in urinary C-peptide) (1.07, 0.92–1.22); duration of diabetes (pre 1 year increase in duration of diabetes) (0.97, 0.91–1.04); basal insulin ratio (per 1% increase in basal insulin ratio) (57.0, 0.06–51119); and once daily insulin in the evening or before bedtime (vs. twice daily insulin, once in the morning and once in the evening or before bedtime) (4.15, 0.54–31.6).

## Discussion

In this study, we examined 64 type 1 diabetic patients receiving basal-bolus insulin therapy (mean HbA1c, 8.7 ± 1.8%). Of these, nocturnal asymptomatic hypoglycemia (<70mg/dL) occurred in 23 patients (35.9%). The occurrence of nocturnal hypoglycemia in these patients was similar in frequency to that reported in other CGM-based studies, where it ranged from 29% to 36% [12–14]. In this regard, the DCCT study previously reported that severe hypoglycemia occurred in 53% of the cases during nighttime [17–19], suggesting that nocturnal hypoglycemia frequently occurs in type 1 diabetes.

Frequent nocturnal hypoglycemia may occur due to 1) the autonomic response to hypoglycemia that becomes attenuated during sleep and 2) the decreased response of counter-regulatory hormones to insulin during sleep [20–22]. Alternatively, it may occur due to the effect of basal insulin peaking during nighttime thus inducing nocturnal hypoglycemia. This study examined patients being treated with long-acting insulin preparations, which last longer than neutral protamine hagedron (NPH) insulin preparations and exert a consistent effect because of their stable absorption. It is suggested, however, that their effects may be less consistent and vary even within the same individuals [23]. The subjects may thus develop nocturnal hypoglycemia, as the effect of insulin given in the evening or before bedtime peaks during nighttime.

In this study, fasting glucose levels were shown to be significantly lower among the hypoglycemic patients (118 ± 35 mg/dL vs. 179 ± 65 mg/dL; P < 0.001), consistently with the results of earlier CGM-based studies in type 1 diabetic patients [13, 14, 24–26], which argue against the concept of the Somogyi effect [15]. On the other hand, the range of post-breakfast glucose elevation, as well as the post-breakfast glucose concentration gradient, was greater among the hypoglycemic patients both at postprandial 1 hour and 2 hours, with their post-breakfast glucose levels seen to surge acutely and rapidly in the presence of nocturnal asymptomatic hypoglycemia.

When the concept of the Somogyi effect was proposed in 1959 [15], NPH insulin was primarily used as basal insulin. Given that NPH insulin is unevenly absorbed subcutaneously and lasts shorter than long-acting insulin, counter-regulatory hormones may have increased soon after onset of nocturnal hypoglycemia with NPH insulin, which, coupled with its shorter duration of action, may have led to rapid increases in glucose levels, thus elevating fasting glucose levels as a consequence. Conversely, the subjects in this study were treated with long-acting insulin preparations lasting longer than NPH insulin. Thus, it is plausible that the long-acting insulin may not have elevated fasting glucose levels, but have elevated glucose levels immediately after breakfast in response to nocturnal asymptomatic hypoglycemia.

The ROC analysis-derived cut-off values for prediction of nocturnal asymptomatic hypoglycemia were: fasting glucose levels, < 135 mg/dL; range of post-breakfast glucose elevation, > 54 mg/dL at postprandial 1 hour; > 78 mg/dL at postprandial 2 hours; and post-breakfast glucose concentration gradient, > 0.90 mg/dL/min at postprandial 1 hour, and > 0.65 mg/dL/min at postprandial 2 hours. The type 1 diabetic patients in this study had a mean HbA1c of 8.7 ± 1.8%, and their mean glucose level estimated as 203 mg/dL [27]. Thus, their actual fasting glucose level 135 mg/dL was shown to be lower than their HbA1c suggested, suggesting that nocturnal hypoglycemia may be suspected when actual fasting glucose levels are lower than those estimated by HbA1c. Study findings also suggest that three glucose measurements a day (before, and 1 hour and 2 hours after breakfast) to determine the range of post-breakfast glucose elevation, as well as post-breakfast glucose concentration gradient, may predict the occurrence of nocturnal asymptomatic hypoglycemia with a close to 65% accuracy, even without having recourse to CGM.

The limitation of the study was that it included a relatively small sample size and that the CGM data obtained in an in-patient setting in the study may have yielded different results than those obtained in an outpatient setting.

Despite this limitation, however, our study results clearly showed the fasting plasma glucose threshold for predicting nocturnal asymptomatic hypoglycemia and suggest that targeting this cut-off value in glycemic control may raise the likelihood of avoiding nocturnal asymptomatic hypoglycemia. Again, it is a key finding from this study that monitoring the range of post-breakfast glucose increase may predict the occurrence of nocturnal asymptomatic hypoglycemia. It is hoped that our data will serve as a reference that helps in formulating insulin therapy which is less associated with hypoglycemia in type 1 diabetic patients.

## Supporting Information

S1 File (XLSX)
